# Assessing the Determinants of Quality of Life and the Impact on HIV Prevention Measures among HIV-Negative and Status-Unknown Young Men Who Have Sex with Men: A Study in Two U.S. Metropolitan Areas

**DOI:** 10.3390/ijerph19020726

**Published:** 2022-01-10

**Authors:** Yu Liu, Savanah Russ, Jason Mitchell, Sarahmona Przybyla, Chen Zhang

**Affiliations:** 1 Department of Public Health Sciences, School of Medicine and Dentistry, University of Rochester, Rochester, NY 14642, USA; savanah_russ@urmc.rochester.edu; 2 Department of Health Promotion and Disease Prevention, Stempel College of Public Health and Social Work, Florida International University, Miami, FL 33199, USA; jamitche@fiu.edu; 3 Department of Community Health and Health Behavior, School of Public Health and Health Professions, University at Buffalo, Buffalo, NY 14214, USA; mona@buffalo.edu; 4 School of Nursing, University of Rochester, Rochester, NY 14642, USA; Chen_zhang@urmc.rochester.edu

**Keywords:** men who have sex with men, quality of life, HIV prevention, psychosocial determinants, mental health, United States

## Abstract

Young men who have sex with men (YMSM) in the United States (U.S.) are disproportionally burdened by HIV and experience adverse social determinants of health. Minimal research has examined quality of life (QoL) and psychosocial/behavioral determinants among HIV-negative or status-unknown YMSM. We conducted a study with YMSM from two U.S. cities to assess their QoL scores, and whether specific QoL domains (e.g., physical, psychological, social, and environment) were associated with their demographics, psychosocial determinants, behavioral risk factors, and HIV prevention measures. Black YMSM, YMSM of low socioeconomic status (below high school education, income < $20,000, and lack of health insurance), and YMSM who did not disclose their sexual orientation had the lowest QoL scores across all domains. Substance use and unprotected anal intercourse were negatively associated with men’s physical/psychosocial health. Housing/food instability and perceived stress were among the strongest predictors of lower QoL in all domains. Higher physical/psychological and environment QoL scores were associated with a higher likelihood of HIV testing and PrEP use. The identification of YMSM within these demographic, behavioral, and psychosocial sub-groups is important for targeted intervention to enhance their well-being and engagement with HIV prevention.

## 1. Introduction

Within the United States, 36,801 individuals were newly diagnosed with HIV in 2019, and over 1.1 million individuals are currently living with HIV [1]. The majority of HIV cases in the U.S. are among those who (1) are living in the south; (2) self-identify as Black/African American or Hispanic/Latinx; and (3) engage in male-to-male sexual contact [1]. Among men who have sex with men (MSM) who were newly diagnosed with HIV, young MSM (YMSM; ages 13 to 34) accounted for over 50% of all new diagnoses of HIV in 2019 [1]. The high HIV burden among this subgroup is of concern given the availability of highly effective prevention measures for protecting against HIV infection including pre-exposure prophylaxis (PrEP) and condom use [2,3].

Beyond the study of sexual risk behaviors associated with HIV acquisition and transmission (e.g., engagement in unprotected anal sex, intravenous drug use) is the need to assess the ways that modifying environmental and social factors may enhance one’s vulnerability to HIV. For example, social support and mental health needs have been identified as salient predictors of engagement in HIV risk behaviors among those who are HIV negative [4]. Both men and women who have good social support have been shown to be less likely to engage in intravenous drug use, have unprotected anal sex, and give/receive money or drugs in exchange for sex. However, those with low social support, as well as high mental health needs, were 50% more likely to engage in the aforementioned HIV risk behaviors [4]. This greater engagement in risky behaviors leads to a greater likelihood of HIV acquisition; the prevalence of HIV among those diagnosed with a serious mental illness is 2–6%, while the prevalence within the general population is lower, at 0.5% [5]. Mental health problems can interfere with HIV prevention behaviors. Within a study of MSM and transgender women, participants that scored higher on a depression scale had lower adherence to PrEP medication [6]. However, uptake of and adherence to PrEP among young men of color have both been shown to be facilitated by support from family and friends through instrumental/logistical support, emotional support, and social interaction [7]. On top of this, key characteristics of support coming from friends and family facilitating PrEP use include closeness, dependability, and homophily (i.e., alikeness) in terms of sexual orientation [7]. Despite variability across demographic groups and HIV serostatus, studies have shown an increase in HIV risk behaviors among MSM with psychosocial health problems [8,9].

One particular psychosocial measure used to evaluate this synergistic relationship between HIV risk and mental health is quality of life. Quality of life (QoL) is defined as an individual’s perception of their position in life in the context of the culture and value systems in which they live, and in relation to their goals, expectations, standards, and concerns [10]. Within the QoL measurement, there are a multitude of domains, including physical health, psychological health, and social relationships. Going beyond traditional measures of well-being, such as life expectancy and cause of death, QoL assesses more granular aspects of an individual’s life, including the prevalence of positive emotions [11]. This measure has been repeatedly used to assess its mediating and moderating effect among those with chronic illnesses, including those diagnosed with HIV. Engelhard et al. identified that in comparison to those diagnosed with rheumatoid arthritis, type 1 diabetes, or type 2 diabetes, individuals living with HIV had poorer mental health–related QoL. Among those with HIV, those who had progressed to AIDS, had long since initiated ART, and had more severe comorbid conditions had poorer physical health–related quality of life [12]. Quality of life has also been shown to differ by HIV status. In a cross-sectional study examining differences in the quality of life between HIV-positive and HIV-negative individuals in South Africa and Zambia, those who were HIV positive reported significantly lower QoL than those who were HIV negative, particularly in the domain of QoL reporting on pain as well as anxiety and depression. However, among those in South Africa, no difference in QoL was observed between those who were HIV negative and those who were HIV positive on ART [13]. While lower QoL has been observed among people living with HIV/AIDS, higher education, annual income, higher CD4 count, and elevated levels of available social support have been found to be positively associated with higher QoL among this population [14,15].

Despite the identification of the positive relationship between QoL and HIV serostatus, significantly less research was conducted among HIV-negative MSM (vs. HIV-positive individuals) to evaluate the performance and determinants of QoL, as well as the plausible impact of QoL on various HIV prevention measures (e.g., PrEP uptake, HIV testing). Among the few published studies, Liu et al. identified how social support among MSM in China, such as receipt of STD testing, health education, and disclosing sexual orientation to a friend or peer, is positively associated with physical and mental QoL [16]. Another study among MSM in China identified how unprotected anal intercourse among MSM is negatively associated with QoL [17]. While these studies provide salient insights into the determinants of QoL among HIV-negative MSM in global contexts, along with the consequences of low QoL on HIV risk behaviors, knowledge remains scarce pertaining to the determinants of QoL and its impact on HIV prevention behaviors among HIV-negative YMSM in the U.S. Given the heightened social stigma and challenges in alleviating the HIV-related burden among YMSM in the U.S., knowledge on aspects of QoL may provide insights in targeting certain YMSM subgroups for facilitating HIV prevention and the promotion of overall mental and physical well-being.

The primary objective of this study was to address the lack of QoL research among HIV-negative YMSM, and to research intervention opportunities to enhance the well-being and engagement with HIV prevention for this population in the U.S. Specifically, we conducted a community-based epidemiologic study to (1) understand the performance and inter-correlation across various domains (e.g., physical, psychological, level of independence, social relationships, environment, spirituality/religion) of QoL; (2) explore significant psychosocial and behavioral factors that may affect various domains of QoL; and (3) understand how various domains of QoL may be associated with risk behaviors (e.g., unprotected sex, substance use) and HIV prevention (e.g., HIV testing, PrEP adoption, condom use) among the study population.

## 2. Materials and Methods

### 2.1. Study Design and Participants

A cross-sectional survey was conducted with YMSM living in Nashville, Tennessee and Buffalo, New York between 2019 May and 2020 May to explore their HIV prevention measures, risk behaviors, and relevant psychosocial determinants of health. Participants were recruited using various convenience sampling methods, including peer referral, flier distribution, social media (e.g., Instagram, Facebook) advertisement, outreach in community events, and venue-based recruitment at local gay bars and HIV prevention centers (i.e., Nashville Council on AIDS, Resources, Education and Support (Nashville CARES), and Evergreen Health Services (EHS) in Buffalo). Participants were considered eligible for enrollment if they: (1) were between 18 and 35 years old, (2) were residents of Nashville or Buffalo, (3) self-identified as cis-gender and gay, bisexual, or a man who had sex with another man in the past 12 months, (4) self-identified as HIV negative or status unknown at the time of enrollment, (5) could read/understand English, and (6) were willing to complete a questionnaire survey and provide informed consent. Institutional Review Boards at the University of Rochester and State University of New York at Buffalo reviewed and approved the study procedure and protocol.

### 2.2. Data Collection and Measures

The details of the data collection procedures have been described elsewhere [18]. In short, we used Research Electronic Data Capture (REDCap) to administer a one-time, 40 min questionnaire survey to enrolled participants for data collection. Participants had the option to complete the survey onsite (i.e., at Nashville CARES or EHS Buffalo) using a provided laptop or tablet, or request the research coordinator to send a secure, unique survey link with an access code to their e-mail address for completing the survey at a different time and location. A gift card with a value of 35 US dollars was provided to each study participant upon completion of the survey to compensate his time and effort for participating.

The REDCap survey contained a variety of measures. Quality of life (QoL) was measured using the WHOQOL-BREF scale, which has been used/validated among MSM and other vulnerable populations in various contexts [17,19,20,21]. The WHOQOL-BREF scale contains 26 items, with 2 items measuring overall QoL and general health, and 24 items that measure QoL across 4 different domains: physical health (PHYSH; 7 items), psychological health (PSYCH; 6 items), social relationships (SOCIR; 3 items), and environment (ENVRT; 8 items). Participants were asked to score each item based on their experiences over the past two weeks on a 5-point Likert scale with response ranges of 1 = “very dissatisfied” or “not at all” or “very poor” to 5 = “very satisfied” or “completely/extremely/an extreme amount” or “very well”. The raw scores for each item were summed for each domain, and then transformed to the 0–100 scale according to the algorithm indicated in the scoring instruction. In this study, the internal consistency (Cronbach’s α) for the physical, psychological, social, and environment domains of WHOQOL-BREF were 0.82, 0.90, 0.86, and 0.92, respectively.

In addition to QoL, other measures in the survey were used to capture participants’ (1) sociodemographics (e.g., age, race, education, employment, income, health insurance, sexual orientation, sexual orientation disclosure to health care providers); (2) behavioral risk factors, including recent (i.e., in the past 12 months prior to the survey) tobacco use, recreational drug use, alcohol binge drinking (see footnotes of the table for details of definitions of respective substance use), and unprotected insertive or receptive anal sex with men; (3) psychosocial determinants and mental health, such as housing stability [18], food insecurity [22], perceived HIV stigma [23], internalized homonegativity [24], perceived social support [25], subjective loneliness [26], suicidal tendency [27], depression [28], anxiety [29], and perceived stress [30]; and (4) HIV prevention uptake and indicators, including self-efficacy toward using condoms [31] and HIV testing [32], as well as general resilience [33], lifetime frequency of HIV testing, and PrEP use (ever/current vs. never).

### 2.3. Statistical Analyses

Because the overall and domain-specific QoL scores were not normally distributed, we computed the median with an interquartile range for all QoL domain scores to be used in all relevant statistical analyses. First, overall and domain-specific QoL scores were computed, followed by Spearman’s correlation analyses to assess the inter-correlations between each of the QoL domain scores. Second, we computed the overall frequency distribution with percentages to describe the sociodemographics and behavioral risk factors for all study participants. We then applied Mann–Whitney U tests or Kruskal–Wallis tests to explore the significant sociodemographic and behavioral correlates for the performance of each domain-specific QoL. Third, we conducted separate multivariable linear regression analyses to assess the association between respective psychosocial/mental health determinants (e.g., housing stability, food insecurity, anxiety, depression, stress, suicidal tendency, perceived HIV stigma, internalized homonegativity, subjective loneliness, perceived social support) and domain-specific QoL scores. Lastly, individual multivariable linear regression or multivariable logistic regression models were fitted to examine the impact of domain-specific QoL performance on each of the selected HIV prevention uptake indicators (e.g., HIV testing frequency, general resilience, PrEP use, and self-efficacy for condom use and testing for HIV). The linearity assumption was checked before fitting the regression models. All multivariable models adjusted for a priori confounders of age, education, income, and sexual orientation. All statistical analyses were conducted using Stata 14.0 (StataCorp LP, College Station, TX, USA).

## 3. Results

### 3.1. QoL Scores and Domain Correlations

The overall median and mean scores of overall QoL and each specific domain (PHYSH, PSYCH, SOCIR, and ENVRT) were reported in Table 1. All domain-specific QoL scores were significantly (*p* < 0.001) correlated with each other, with ENVRT and PSYCH showing the strongest correlation (*ρ* = 0.788), followed by the correlation of SOCIR–PSYCH (*ρ* = 0.775), ENVRT–PHYSH (*ρ* = 0.769), ENVRT–SOCIL (*ρ* = 0.753), and PHYSH–PCYCH (*ρ* = 0.750).

### 3.2. Sociodemographics and QoL

We enrolled a total of 415 study participants, of which 374 completed the survey in full. Of the 374 study participants, 57.6% were 25–35 years of age, 60.2% were non-Hispanic Black, 79.6% had above a high school education, 70.1% were employed, 41.5% had less than $20,000 in annual income, 81.3% had health insurance, and 77.0% self-identified as gay/homosexual (Table 2). YMSM who self-identified as bisexual (*p* = 0.006) or non-Hispanic Black (*p* = 0.014) reported significantly lower ENVRT scores compared to YMSM who were white or another race. Having at least a college degree (vs. not), >$40,000 annual income (vs. ≤$40,000), and health insurance (vs. not) were significantly (*p* < 0.05) associated with higher scores across all QoL domains. YMSM who disclosed their sexual orientation to a healthcare professional were more likely to report having higher PHYSH (*p* = 0.019) and PSYCH (*p* = 0.011) scores compared to YMSM who did not disclose their sexual orientation.

### 3.3. Behavioral Risk Factors and QoL

Table 3 shows the prevalence of substance use, sexual behaviors, and their associations with QoL among YMSM in the current study. Many of the study participants reported having used tobacco (74.4%) and recreational drugs (81.0%), and binge drinking alcohol (71.2%), whereas a lower proportion of them reported alcohol/drug use before sex (40.4%) and engagement in unprotected insertive/receptive anal sex in the past 12 months (59.6%). Participants who used substances and/or engaged in insertive/receptive anal sex without a condom were significantly (*p* < 0.05) associated with reporting lower PHYSH and PSYCH scores compared to those who did not.

### 3.4. Psychosocial and Mental Health Determinants of QoL

The results of multivariable analyses of psychosocial and mental health factors on QoL are shown in Table 4. Despite the variation in effect sizes and different levels of statistical significance, we found that a variety of adverse psychosocial/mental health indicators were negatively associated with all domains of QoL, including depression, anxiety, stress, perceived HIV stigma, internalized homonegativity, suicidal tendency, loneliness, and food insecurity. Food insecurity demonstrated the strongest negative impact on overall QoL/general health (β = −2.802; *p* < 0.001), PHYSH (β = −2.352; *p* < 0.001), and ENVRT (β = −3.153; *p* < 0.001); suicidal tendency was the most influential factor on PSYCH (β = −1.853; *p* < 0.001); and perceived stress had the strongest influence on SOCIL (β = −2.338; *p* < 0.001). Protective factors, such as stable housing and perceived social support, were found to significantly improve performance across all QoL domains, with housing stability showing the strongest buffering effect on ENVRT (β = 2.654; *p* < 0.001) and perceived social support on SOCIL (β = 0.739; *p* < 0.001).

### 3.5. Impact of QoL on HIV Prevention Measures

As shown in Table 5, all QoL domains were positively associated (*p* < 0.001) with HIV testing self-efficacy (HTSE) and general resilience (GR) among the study participants, with PSYCH showing the strongest association with HTSE (β = 0.147) and GR (β = 0.275). PHYSH, PSYCH, and ENVRT were significantly (*p* < 0.05) associated with lifetime HIV testing frequency and a higher likelihood of PrEP use. Lastly, we found overall QoL/health (β = 0.052; *p* < 0.001), PHYSH (β = 0.047; *p* < 0.01), and PSYCH (β = 0.035; *p* < 0.05) were positive indicators of condom use self-efficacy.

## 4. Discussion

The present analysis extends the literature on QoL among YMSM in the U.S. by providing a snapshot about YMSM’s QoL and how the QoL domains (physical health, psychological health, social relationships, and environment) were associated with a variety of modifiable psychosocial determinants of health. Our study also identified the relationship between QoL domains and the impact they have on YMSM’s engagement in substance use, sexual risk behaviors, and HIV prevention. Overall, YMSM’s QoL varied by domain, and notable differences were found in certain subgroups within our sample (as evident in Table 2, Table 3 and Table 4), as well as in related HIV prevention measures. In comparison with previously reported QoL among MSM living in China reporting having had unprotected anal intercourse (UAI), the QoL scores found in our study were similar for the psychological and physical health domains. However, domain scores among MSM in China reporting not having engaged in UAI were higher for both physical and psychological health [17]. These differences may stem from the high percentage (~60%) of YMSM in our study who reported having engaged in unprotected insertive or receptive anal intercourse, suggesting that engagement in UAI is a robust predictor of reported QoL among MSM. Finally, the strong and significant correlations between the domains of psychological health and social relationships as well as environment are consistent with prior findings [4,34,35]. Social support has been shown to be an important mediator of the association between mental health needs and HIV risk behaviors, particularly among males [4]. Environment, including the persistence of low-quality and crowded housing, has consistently been found to be associated with poorer mental health [34,35]. Further studies examining (1) scores of MSM across each QoL domain and (2) the mechanism by which these domains inter-correlate with one another would provide valuable insight into potential drivers of QoL scores.

Our study identified indicators of lower socioeconomic status (SES), including education, annual income, and health insurance, as predictors of lower QoL scores across almost all domains, particularly psychological health. One possible explanation for this association surrounds an increase in psychological distress among low SES populations. Perceived stress has previously been identified as a strong mediator between socioeconomic status and health status, particularly among racial and ethnic minorities [36,37]. Low socioeconomic status has also been shown to be a moderating factor for the association between poor social relations and adverse health behaviors, which ultimately has implications for mental health outcomes [38]. These identified increases of psychological distress may contribute to subsequently reported perceptions of low quality of life across these respective domains. This information stands to contribute to future intervention efforts as YMSM in low socioeconomic status sub-groups may be more likely to engage in HIV prevention and care with financial incentives. Along with socioeconomic status, individuals who identify as Black are predisposed to systematic and fundamental societal inequities that ultimately have highly negative and consequential effects on their social environment. Thorpe et al. identified that disparities in health behaviors between African American and white men, such as being physically inactive and smoking, were eliminated when environment and socioeconomic status were adjusted for, suggesting race has a strong impact on an individual’s experienced environment [39]. Finally, sexual orientation and disclosure of sexual orientation were also found to be predictors of the environment score. This result is consistent with prior findings of higher levels of lifetime and day-to-day discrimination among those identifying as homosexual or bisexual [40]. It is important to acknowledge the higher physical, psychological, and environmental scores among those who reported disclosing their sexual orientation to a healthcare professional given the potential negative implications of concealment on mental and physical health. Such implications include an increased likelihood of depression and anxiety symptoms, as well as a lack of effective screening by health care providers given the omitted pertinent information regarding risk factors for particular cancers [41,42].

Behavioral risk factors, including substance use and sexual risk behaviors, were found to be negatively associated with reported physical and psychological health QoL domains. Substance use, including the use of tobacco, alcohol, and recreational drugs, has been previously linked to an increased risk of inadvertent injuries across all ages, including vehicular accidents and damaging falls [43]. Substance use has also been linked to higher rates of a variety of medical conditions, such as cancer, sexually transmitted infections, and heart disease. Along with clinical illnesses, substance use has been linked to an increased risk of bipolar, anxiety, and antisocial personality disorders [43]. YMSM who currently or historically have used tobacco, alcohol, or recreational drugs should be identified as having a greater risk for reporting lower physical and psychological health, and they should be targeted for early interventions to mitigate such risks, including rigorous screening and early treatment. This is particularly salient for this population as MSM with mental health diagnoses are at a greater risk for HIV acquisition [44]. Sexual risk behaviors, such as unprotected insertive or receptive anal intercourse, have been linked to lower support for safer sex in the broader gay community as well as concerns about stigmatization [45]. These risk-taking behaviors have also been found to be associated with symptoms of anxiety among MSM [46,47]. These results, along with prior literature, suggest engagement in high-risk sexual behaviors results in lowered perceived physical and psychological health, both through the risk of disease acquisition and heightened anxiety surrounding this increased risk. However, interventions targeted at the community level, including social activities designed to educate participants on HIV risks and prevention, have been shown to reduce the risk of HIV transmission and improve mental health outcomes. The identification of YMSM who engage in both substances use as well as sexual risk behaviors, along with the implementation of educational interventions, can mitigate low perceived physical and mental health, enhancing the uptake of HIV prevention measures among this population.

When evaluating the impact of psychosocial determinants on QoL domain scores, our study identified that all determinants, including food and housing security, external and internal homonegativity, social support, and mental health were significant predictors of reported QoL. Food and housing insecurity have previously been shown to be associated with an increased risk of psychological distress, along with infrequent access to health insurance and ambulatory care resulting in lower physical health [48,49,50]. Individuals in public housing have also reported environmental barriers to achieving a state of well-being and making healthy life choices, compounding the association between housing instability and physical health [51]. Social support has been identified as a factor that can improve QoL among MSM populations, as well as lower the prevalence of life chaos and rates of depression [16,52,53]. Future HIV prevention efforts may be greatly improved by incorporating peer support to increase uptake and adherence. Suicidal tendencies and ideation have previously been found to be high among MSM, particularly among those who are HIV positive. Suicidal behavior has also been found to be more highly reported among MSM who are HIV positive, experiencing perceived minority stress, or have high levels of psychological distress [54,55,56]. The implementation of screening for suicidal tendencies concurrently with HIV prevention and care services stands to greatly improve reported physical and psychological QoL domains among this population. Finally, the relationship between mental health outcomes, such as depression and anxiety, and lower reported QoL domain scores is well-established [57]. This relationship may be explained by the modifying effect that perceived stigma has on psychological distress as well as health outcome reporting [58,59]. Given that 70% of our study participants are YBMSM, the impact of stigma on psychological health, either from being gay or possibly having HIV, may be compounded by the discrimination faced as a minority population. Efforts to improve reported QoL scores should be tailored to the stigma faced by the MSM population, as well as to specific racial and ethnic minority MSM populations for the most effective outreach.

Finally, our study identified the impact that QoL domains, such as physical health, psychological health, and environment, had on HIV prevention measures. The strong association between higher psychological health scores and engagement in HIV prevention measures may stem from the previously established high correlation between mental health and social support, and between social support and engagement in HIV prevention. Given that social and peer support among populations at high risk for HIV acquisition have been shown to increase engagement with HIV testing and PrEP use, the relationship between psychological health and HIV prevention measures may be mediated by levels of support [60,61,62,63]. Further investigation into the role that social support may have on this pathway is warranted. Subsequently, the association between environment domain scores and PrEP use is consistent with the literature when considering the environment to include access to adequate and sustainable health care [64]. The consideration of such QoL scores when targeting groups of YMSM with low engagement in HIV care and prevention is important to better access at-risk sub-groups.

To the best of our knowledge, this is the first study to examine the performance of various QoL domains and their associations with psychosocial determinants, behavioral factors, and HIV prevention among YMSM in the U.S. There are several limitations to our study, the first of which being that participants were recruited from two U.S. cities using convenience sampling. Along with a relatively small sample size, this limits the generalizability of our findings. Secondly, our results may be subject to recall and social desirability biases; despite the use of computer-assisted self-interview, our questionnaire required participants to self-report sensitive content including sexual history, substance use, and HIV prevention measures. Third, given that our study design was cross-sectional, the inference we draw from our findings regarding the relationship between QoL reporting and psychosocial determinants as well as engagement with HIV prevention is limited given the lack of temporality. Finally, we were only able to assess information on the psychosocial determinants of mental health within the last 4 weeks, which limits our understanding and contribution to the establishment of a theoretical framework for the associations of interest in this study.

## 5. Conclusions

QoL domains including psychological health and social relationships/environment are strongly correlated. YMSM of low socioeconomic status, Black YMSM, and those identifying as bisexual are at a greater risk for reporting lower QoL scores. Behavioral risk factors and psychosocial determinants including drug/alcohol use, sexual risk taking, housing/food insecurity, perceived social support, suicidal tendency, and perceived stress all influence reported QoL among YMSM. The reporting of QoL scores ultimately has salient implications for engagement in HIV prevention behaviors, including HIV testing and PrEP use. The identification and targeting of YMSM who meet these characteristics within HIV care and prevention efforts may lead to the interruption of mechanisms driving low engagement with HIV prevention among this population.

## Figures and Tables

**Table 1:** Bivariate associations and performance of quality-of-life domain scores among young men who have sex with men in two U.S. cities (*N* = 347).

QoL Domains	Mean Scores (SD)	Median Scores (IQR)	Spearman’s Correlation Coefficients				
			1	2	3	4	5
1. Overall QoL and general health	74.5 (21.9)	75.0 (62.5–87.5)	1				
2. Physical health	68.5 (19.7)	71.4 (57.1–82.1)	0.525 ***	1			
3. Psychological health	59.4 (19.8)	62.5 (45.8–70.8)	0.624 ***	0.750 ***	1		
4. Social relationships	58.7 (26.4)	58.3 (41.7–75.1)	0.549 ***	0.667 ***	0.775 ***	1	
5. Environment	65.0 (23.5)	68.8 (53.1–81.3)	0.556 ***	0.769 ***	0.788 ***	0.753 ***	1

Note: *** *p* < 0.001; QoL, quality of life; SD, standard deviation; IQR, interquartile range.

**Table 2:** The associations of quality-of-life domains with sociodemographic characteristics among young men who have sex with men in two U.S. cities (*N* = 347).

	Total (*N* = 347)	QoL Domains (Median, IQR)				
Sociodemographics	*n* (%)	Overall QoL and Health	Physical Health	Psychological Health	Social Relationships	Environment
Age (years)						
18–24	147 (42.4)	76.1(62.5–87.5)	72.6(53.6–82.1)	63.7 (45.8–70.8)	58.2 (41.7–75)	68.8 (50–81.3)
25–35	200 (57.6)	75 (62.5–100)	71.4 (57.1–82.1)	62.5 (50–70.8)	59.3 (41.2–74.6)	71.9 (56.3–78.1)
*p*-value		0.572	0.329	0.381	0.786	0.294
Race/ethnicity						
Non-Hispanic white	109 (31.4)	75 (62.5–87.5)	71.4 (60.7–82.1)	62.5 (50–70.8)	58.3 (50–75)	71.8 (62.5–81.2)
Non-Hispanic Black	209 (60.2)	73 (62.5–100)	69.8 (50–82.1)	62.5 (45.8–75)	58.3 (41.7–75)	65.6 (50–78.1)
Other *	29 (8.4)	73.5 (62.5–87.5)	71.4 (57.1–82.1)	58.3 (41.7–66.7)	66.7 (50–75)	68.8 (53.1–78.2)
*p*-value		0.069	0.298	0.362	0.898	0.014
Education						
High school or lower	71 (20.4)	70 (50–87.5)	60.7 (46.4–82.1)	54.1 (41.7–70.8)	50 (41.7–75)	59.3 (40.6–78.1)
Some college	138 (39.8)	72 (62.5–100)	64.3 (50–78.6)	58.3 (45.8–70.8)	62.5 (50–75)	62.5 (50–75)
College and above	138 (39.8)	75 (75–87.5)	75 (67.8–85.7)	66.7 (54.1–70.8)	66.6 (33.3–83.3)	71.8 (65.6–81.3)
*p*-value		0.011	<0.001	0.004	0.009	<0.001
Employment						
Employed	243 (70.1)	75 (62.5–87.5)	71.4 (57.1–82.1)	62.5 (45.8–70.8)	58.3 (41.7–75)	68.8 (56.3–81.3)
Unemployed	51 (14.7)	75 (50–100)	78.6 (53.6–85.7)	62.5 (45.8–83.3)	66.7 (50–75)	68.8 (50–84.3)
College students	53 (15.2)	75 (62.5–87.5)	64.2 (57.1–75)	62.5 (45.8–70.8)	58.3 (33.3–75)	68.8 (56.3–78.1)
*p*-value		0.842	0.479	0.839	0.140	0.965
Annual income						
<$20,000	144 (41.5)	75 (62.5–87.5)	67.8 (53.5–82.1)	60.4 (45.8–70.8)	58.3 (41.7–75)	65.6 (46.9–78.1)
$20,000–$40,000	132 (28.1)	75 (62.5–87.5)	71.4 (55.3–82.1)	62.5 (50–70.8)	58.3 (41.7–75)	68.8 (56.3–75)
>$40,000	71 (20.4)	75 (75–100)	71.4 (60.7–89.2)	62.5 (54.2–75)	66.6 (58.3–83.3)	75 (59.4–90.6)
*p*-value		0.469	0.046	0.008	0.017	<0.001
Health insurance						
Uninsured	65 (18.7)	71 (50–87.5)	57.1 (46.4–78.6)	54.2 (45.8–66.7)	50 (33.3–66.7)	53.1 (37.5–65.6)
Insured	282 (81.3)	76.5 (62.5–87.5)	71.4 (60.7–82.1)	62.5 (50–70.8)	58.3 (50–75)	71.9 (59.4–81.2)
*p*-value		0.013	<0.001	<0.001	0.003	<0.001
Sexual orientation						
Homosexual	267 (77.0)	75 (62.5–87.5)	71.4 (57.1–82.1)	62.5 (50–70.8)	58.3 (41.7–75)	71.9 (56.2–81.3)
Heterosexual	32 (9.2)	81.3 (37.5–100)	66.1 (46.4–78.6)	66.7 (45.8–72.9)	70.8 (37.5–91.6)	75 (42.1–82.8)
Bisexual	48 (13.8)	75 (62.5–87.5)	67.8 (48.2–76.7)	54.2 (41.7–66.7)	58.3 (50–75)	59.4 (50–68.7)
*p*-value		0.886	0.05	0.035	0.505	0.006
Sexual orientation disclosure to healthcare professionals						
No	82 (23.6)	75 (62.5–87.5)	66.1 (42.9–78.6)	58.3 (41.6–78.3)	58.3 (50–75)	60.9 (40.6–78.1)
Yes	265 (76.4)	75 (62.5–100)	71.4 (57.1–82.1)	62.5 (50–70.8)	58.3 (25–75)	68.8 (56.2–81.2)
*p*-value		0.213	0.019	0.011	0.141	0.016

Note: IQR, interquartile range; QoL, quality of life. * Including Hispanic/Latino, Asian, and not sure.

**Table 3:** Behavioral risk factors and their associations with quality of life among young men who have sex with men in two U.S. cities (*N* = 347).

	Total (*N* = 347)	QoL Domains (Median, IQR)				
Behavioral Risk Factors	*n* (%)	Overall QoL and Health	Physical Health	Psychological Health	Social Relationships	Environment
Current tobacco use in the past 12 months ^‡^						
No	89 (25.6)	83.5 (62.5–87.5)	72.6 (57.1–85.7)	66.7 (54.1–79.2)	66.7 (41.7–91.6)	72.6 (59.4–84.3)
Yes	258 (74.4)	75 (62.5–87.5)	67.9 (53.6–82.1)	62.5 (45.8–70.8)	58.3 (41.7–75)	68.7 (50–78.1)
*p*-value		0.101	0.014	0.005	0.093	0.056
Recreational drug use in the past 12 months ^†^						
No	66 (19.0)	75 (62.5–87.5)	71.4 (57.1–82.1)	67.5 (45.8–70.8)	58.3 (50–75)	71.8 (53.1–81.2)
Yes	281 (81.0)	75 (62.5–87.5)	64.3 (53.5–78.5)	61.7 (45.8–70.8)	58.3 (41.7–75)	62.5 (53.2–75)
*p*-value		0.945	0.019	0.029	0.916	0.094
Alcohol binge in the past 12 months ^※^						
No	100 (28.8)	75 (62.5–93.7)	73.2 (57.1–85.7)	66.7 (54.1–81.3)	66.7 (50–75)	71.8 (59.4–76.6)
Yes	247 (71.2)	75 (62.5–87.5)	67.5 (57.1–82.1)	62.5 (45.8–70.8)	58.3 (41.7–75)	68.7 (53.1–81.3)
*p*-value		0.358	0.008	0.013	0.069	0.361
Alcohol/drug use before sex in the past 12 months						
No	207 (59.6)	75 (62.5–87.5)	71.4 (57.1–82.1)	62.5 (50–75)	58.3 (41.7–75)	71.8 (56.2–81.2)
Yes	140 (40.4)	75 (62.5–87.5)	67.8 (53.6–82.1)	58.3 (45.8–70.8)	58.3 (41.7–75)	65.6 (51.6–75)
*p*-value		0.126	0.045	0.036	0.512	0.05
Unprotected insertive/receptive anal sex with men in the past 12 months						
No	137 (40.4)	87.5 (75–100)	73.4 (64.2–85.7)	66.7 (33.3–70.8)	58.3 (41.7–75)	71.8 (56.2–81.2)
Yes	202 (59.6)	75 (62.5–87.5)	67.8 (53.6–82.1)	62.5 (45.8–70.8)	58.3 (41.7–75)	68.7 (50–78.1)
*p*-value		0.009	0.042	0.032	0.297	0.232

Note: IQR, interquartile range; QoL, quality of life. **^‡^** Tobacco use, including intake or smoking (even a puff) the following products: regular cigarette, e-cigarette, bidi, cigar, hookah, pipe, dip, chewing tobacco, dissolvable, snuff, or snus. **^†^** Recreational drug use: self-report intake of rush poppers (alkyl nitrites), crystal meth (methamphetamine), marihuana, hallucinogens (ketamine, LSD, PCP, etc.), cocaine, heroin or other opioids, Magu (a mixture of methamphetamine and caffeine), opium, triazolam tablets (benzodiazepines), or ecstasy (3,4-methylenedioxymethamphetamine, MDMA). ^※^ Binge drinking is defined as having six or more standard drinks (i.e., 12 ounces (one can) of beer (5% alcohol), 6 ounces (1 glass) of wine (12% alcohol), 1.5 ounces (1 shot) of liquor (40% alcohol)) during a drinking occasion.

**Table 4:** Multivariable analysis of the associations of psychosocial factors and mental health burdens with quality of life among young men who have sex with men (*N* = 347).

	Quality of Life				
Psychosocial Determinants ^†^	Overall QoL/Health β (SE)	Physical Health β (SE)	Psychological Health β (SE)	Social Relationships β (SE)	Environment β (SE)
Housing stability	1.432 (0.419) **	2.134 (0.364) ***	1.101 (0.379) **	1.093 (0.501) *	2.654 (0.432) ***
Food insecurity	−2.802 (0.503) ***	−2.352 (0.454) ***	−0.903 (0.471)	−1.897 (0.623) **	−3.153 (0.535) ***
Perceived HIV stigma	−0.397 (0.125) **	−0.224 (0.114) *	−0.009 (0.115)	−0.459 (0.151) **	−0.063 (0.136)
Internalized homonegativity	−1.338 (0.267) ***	−0.871 (0.244) ***	−0.654 (0.247) **	−1.086 (0.327) **	−0.728 (0.293) *
Perceived social support	0.447 (0.051) ***	0.498 (0.043) ***	0.528 (0.042) ***	0.739 (0.055) ***	0.623 (0.050) ***
Loneliness	−1.302 (0.221) ***	−0.509 (0.207) *	−0.065 (0.209)	−1.091 (0.273) ***	−0.187 (0.247)
Suicidal tendency	−2.411 (0.348) ***	−1.809 (0.319) ***	−1.853 (0.327) ***	−2.267 (0.433) ***	−1.746 (0.386) ***
Anxiety	−1.167 (0.193) ***	−0.916 (0.176) ***	−0.231 (0.182) **	−0.747 (0.241) **	−0.481 (0.215) *
Depression	−1.062 (0.146) ***	−0.949 (0.132) ***	−0.437 (0.139) **	−0.941 (0.182) ***	−0.545 (0.166) **
Perceived stress	−1.512 (0.188) ***	−1.668 (0.161) ***	−1.586 (0.164) ***	−2.338 (0.215) ***	−1.649 (0.201) ***

Note: SE, standard error; * *p* < 0.05; ** *p* < 0.01; *** *p* < 0.001. ^†^ Separate multiple linear regression model was used to evaluate each psychosocial and mental health factor with each QoL domain, respectively. Each model individually adjusted for a priori sociodemographic confounders, including age, education, income, and sexual orientation.

**Table 5:** Multivariable analysis of the associations between quality-of-life performance and HIV prevention measures among young men who have sex with men (*N* = 347).

			HIV Prevention Measures		
QoL Domains	Condom Use Self-Efficacy (Continuous) β (SE)	HIV Testing Self-Efficacy β (SE)	General Resilience β (SE)	Frequency of HIV Testing during Lifetime β (SE)	PrEP Use (Ever/Current vs. Never) aOR (95% CI)
Overall QoL/health	0.052 (0.014) ***	0.106 (0.026) ***	0.192 (0.019) ***	0.001 (0.001)	1.01 (1.00–1.02) *
Physical health	0.047 (0.016) **	0.146 (0.029) ***	0.246 (0.021) ***	0.004 (0.001) **	1.03 (1.02–1.05) ***
Psychological health	0.035 (0.015) *	0.147 (0.028) ***	0.275 (0.019) ***	0.005 (0.001) ***	1.03 (1.02–1.04) ***
Social relationships	0.022 (0.011)	0.093 (0.022) ***	0.174 (0.015) ***	0.001 (0.001)	1.01 (1.00–1.02)
Environment	0.023 (0.013)	0.101 (0.024) ***	0.203 (0.017) ***	0.003 (0.001) **	1.02 (1.01–1.04) ***

Note: QoL, quality of life; SE, standard error; aOR, adjusted odds ratio; CI, confidence interval; * *p* < 0.05; ** *p* < 0.01; *** *p* < 0.001. Each model individually adjusted for a priori sociodemographic confounders, including age, education, income, and sexual orientation.

## Data Availability

The data presented in this study are available on request from the corresponding author. The data are not publicly available due to privacy concerns and protection of sensitive information from the research subjects.

## Abbreviations

HIV—Human immunodeficiency virus; MSM—Men who have sex with men; YMSM—Young men who have sex with men; PrEP—Pre-exposure prophylaxis; QoL—Quality of life; ART—Anti-retroviral therapy; Nashville CARES—Nashville Council on AIDS, Resources, Education and Support; EHS—Evergreen Health Services; REDCap—Research Electronic Data Capture; WHOQOL-BREF—The World Health Organization Quality of life questionnaire for HIV Brief Version; PHYSH—Physical health; PSYCH—Psychological health; SOCIR—Social relationships; ENVRT—Environment; HTSE—HIV testing self-efficacy; GR—General resilience; UAI—Unprotected anal intercourse; SES—Socioeconomic status.
